# The therapeutic potential of triptolide and celastrol in neurological diseases

**DOI:** 10.3389/fphar.2022.1024955

**Published:** 2022-10-19

**Authors:** Yueran Cui, Xuejiao Jiang, Juan Feng

**Affiliations:** ^1^ Department of Neurology, Shengjing Hospital of China Medical University, Shenyang, Liaoning, China; ^2^ Department of Otolaryngology Head and Neck Surgery, Shengjing Hospital of China Medical University, Shenyang, Liaoning, China

**Keywords:** neurological diseases, traditional Chinese medicine, triptolide, celastrol, neuroprotection, toxicity, derivatives

## Abstract

Neurological diseases are complex diseases affecting the brain and spinal cord, with numerous etiologies and pathogenesis not yet fully elucidated. *Tripterygium wilfordii Hook. F.* (TWHF) is a traditional Chinese medicine with a long history of medicinal use in China and is widely used to treat autoimmune and inflammatory diseases such as systemic lupus erythematosus and rheumatoid arthritis. With the rapid development of modern technology, the two main bioactive components of TWHF, triptolide and celastrol, have been found to have anti-inflammatory, immunosuppressive and anti-tumor effects and can be used in the treatment of a variety of diseases, including neurological diseases. In this paper, we summarize the preclinical studies of triptolide and celastrol in neurological diseases such as neurodegenerative diseases, brain and spinal cord injury, and epilepsy. In addition, we review the mechanisms of action of triptolide and celastrol in neurological diseases, their toxicity, related derivatives, and nanotechnology-based carrier system.

## 1 Introduction

Neurological diseases are disorders affecting the brain and spinal cord, which are caused by many endogenous and exogenous factors (Mehndiratta and Aggarwal, 2021). These functional or organic disorders cause patients to exhibit many corresponding clinical symptoms, such as dementia, pain and muscle strength disorders, which place a tremendous burden on the patients and society (Borsook, 2012). With the accelerated aging of the population, the incidence of neurological diseases is gradually increasing, and it is becoming a global problem. However, due to the complexity of neurological diseases, the pathogenesis of neurological diseases is not yet clear and effective treatments are limited (Yang et al., 2022a). Studies have shown that many factors are involved in the pathogenesis of neurological diseases (Liu Q. et al., 2022; Delport and Hewer, 2022; Wu et al., 2022). A growing number of studies have suggested that inflammation and immunity play an important role in the development of neurological diseases (Perry et al., 2010; Schiavone and Trabace, 2017; Jiang et al., 2018). Therefore, the investigation of the role of inflammation and immunity in neurological diseases, as well as the related therapeutic approaches, may contribute to the effective treatments of neurological diseases.

**FIGURE 1:**
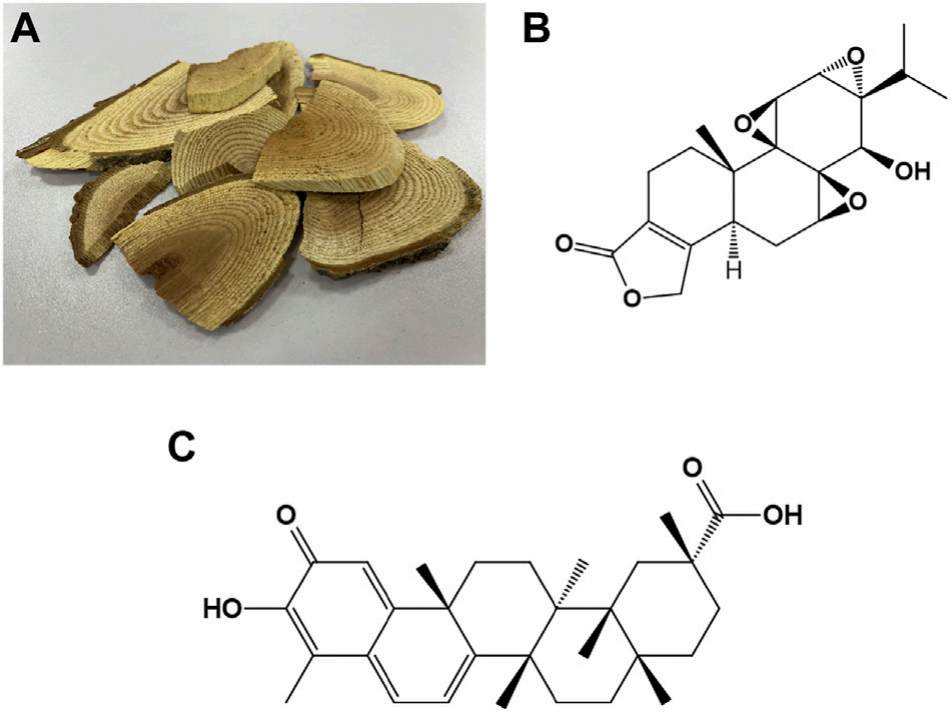
The photograph of woody roots of Tripterygium wilfordii Hook. f. and the chemical structures of triptolide and celastrol **(A)** The photograph of woody roots of Tripterygium wilfordii Hook. f. **(B)** Chemical structure of triptolide. **(C)** Chemical structure of celastrol.

*Tripterygium wilfordii Hook. F* (TWHF) is a perennial woody vine, which grows mainly in Eastern and Southern China, Japan, and Korea (Brinker et al., 2007). As a classical traditional Chinese medicine, TWHF has a history of over 2,000 years of medicinal use (Li and Hao, 2019). TWHF has a wide range of mechanisms of action and has been used in a variety of diseases, especially autoimmune and inflammatory diseases such as rheumatoid arthritis and dermatomyositis (Wang et al., 2016). Currently, more than 100 components have been extracted from TWHF, of which triptolide and celastrol play an important role in the core bioactivity of TWHF, the anti-inflammatory and antioxidant (Figure 1) (Li and Hao, 2019). Triptolide is an important diterpenoid isolated from TWHF and was first extracted for medicinal research in 1972 (Kupchan et al., 1972). Celastrol is derived from a wide variety of natural plants and accounts for a large percentage of the extracts of the roots of TWHF (Lange et al., 2017). Studies have found that triptolide and celastrol are therapeutically effective in preclinical and clinical studies in a variety of diseases, which may be related to their anti-inflammatory and antioxidant effects (Gao et al., 2021; Schiavone et al., 2021). Because of their small molecular weight and lipophilicity, they can cross the blood-brain barrier and work in the brain, so they have been studied in many neurological diseases (Cleren et al., 2005; Lu et al., 2010; De Angelis et al., 2020), and their roles in neurological diseases are the focus of this review (Table 1).

**TABLE 1:** Effects of triptolide and celastrol on neurological diseases.

Disease	Component	*In vivo*/*In vitro*	Effects	References
Alzheimer’s disease	Triptolide	*in vivo*; Aβ 1-40-induced AD rats	attenuates the degeneration of dendritic spines in hippocampal neurons	Wan et al. (2014)
		*in vivo*; APP/PS1 double transgenic AD murine model	improves cognitive function, accompanied by reduced neuroinflammation and Aβ deposition	Cheng et al. (2014)
		*in vivo*; APP/PS1 double transgenic AD murine model	reduces astrocyte proliferation and microglia activation in the hippocampal region	Li et al. (2016)
		*in vivo*; APP/PS1 double transgenic AD murine model	improves spatial memory deficits by inhibiting of BACE1	Wang et al. (2014)
		*in vivo*; APP/PS1 double transgenic AD murine model	improves spatial memory deficits by inhibiting inflammatory responses and MAPKs activity	Cui et al. (2016)
		*in vitro*; SH-SY5Y cell lines	exerts neuroprotective effects by inhibiting CXCR2 activity and reducing Aβ production	Wang et al. (2013b)
		*in vitro*; Aβ 1-42-treated cultured rat microglia	exerts protective effects by inhibiting TNF-α and IL-1β expression levels	Jiao et al. (2008)
		*in vitro*; glutamate-stimulated PC12 cells	inhibits the level of ROS, attenuating apoptosis	He et al., 2003; Gu et al., 2004
		*in vitro*; Aβ 25-35-treated differentiated PC12 cells	promotes autophagy and inhibits oxidative stress, exerting neuroprotective effects	Xu et al., 2015; Xu et al., 2016
		*in vitro*; Aβ 1-42-treated differentiated PC12 cells	nanoparticles loaded with triptolide reduces oxidative stress and inhibits cytotoxicity	Jia et al. (2021)
		*in vitro*; primary astrocytes from rats	increases synthesis and release of nerve growth factor	Xue et al. (2007)
		*in vitro*; hippocampal neurons	increases the expression of synaptophysin	Nie et al. (2012)
		*in vivo*; APP/PS1 double transgenic AD murine model	increased hippocampal neuroligin-1 expression through epigenetic mechanisms	Lu et al. (2019)
	Celastrol	*in vivo*; LPS rat model	improves performance in memory and learning activity tests	Allison et al. (2001)
		*in vivo*; double transgenic Tg PS1/APPsw AD mice	attenuates the accumulation of pathological plaque and microglial activation	Paris et al. (2010)
		*in vivo*; diabetes mellitus rat model	improves cognitive function and decreases amyloid substance	Liao et al. (2018)
		*in vivo*; Aβ 25-35-microinjected rats	improves learning and memory deficits by inhibiting NF-kB activity, improving synaptic function and increasing glucose metabolism	Xiao et al. (2021)
		*In vivo* and *vitro*; P301S microtubule associated protein tau mice and 3XTg mice, N2a cells	activates transcription factor EB-mediated autophagy and lysosome biogenesis, reduce the accumulation of neurofibrillary tangles, thereby attenuating disease severity	Yang et al. (2022b)
		*in vitro*; human monocytes and macrophages, microglia, endothelial cells	inhibits the production of TNF-α and IL-1β by human monocytes and macrophages, the expression of MHC II molecules of microglia, and the production of inducible nitric oxide in endothelial cells	Allison et al. (2001)
		*in vitro*; HEK293 cells	attenuates NF-kB activity	Paris et al. (2010)
		*in vitro*; 7 W CHO cells overexpressing wild-type human APP	inhibits amyloid-β production by inhibiting BACE-1	Paris et al. (2010)
		*in vitro*; IMR-32 cells	exerts neuroprotective effects by inhibiting IKK	Veerappan et al. (2017)
		*in vitro*; LPS-treated H4-APP cells	celastrol inhibits the production of Aβ, attenuates NF-kB activity and suppresses COX-2 expression. In addition, celastrol increases the expression of Hsp70 and Bcl-2	Zhao et al. (2014)
		*in vitro*; Aβ 1-42-treated SH-SY5Y cells	no effect on the expression of Hsp70, while inhibits the expression of Hsp90	Cao et al. (2018)
Parkinson’s disease	Triptolide	*in vivo*; LPS rat model	exerting neuroprotective effects by protecting dopaminergic neurons and reducing the expression of pro-inflammatory cytokines (TNF-α and IL-1β)	Zhou et al. (2005)
		*in vivo*; LPS rat model	protecting dopaminergic neurons and inhibiting microglia activation	Li et al. (2006b)
		*in vivo*; MPP + -induced rat model	improving behavioral performance by protecting dopaminergic neurons and inhibiting microglial activation	Gao et al. (2008)
		*in vitro*; LPS-induced primary mesencephalic neuron/glia mixed culture	decreases [3H]dopamine uptake and loss of tyrosine hydroxylase-immunoreactive neurons, inhibits microglial activation, and attenuates TNF-α and NO production	Li et al. (2004)
		*in vitro*; MPP + -induced primary mesencephalic neurons	tripchlorolide; promotes axonal elongation and protects dopaminergic neurons, as well as increases *BDNF* mRNA expression	Li et al. (2003)
		*in vivo*; partially lesioned PD rat model	tripchlorolide; protects dopaminergic neurons and inhibits the overproduction of TNF-α and IL-2	Cheng et al. (2002)
		*in vivo*; MPTP-induced PD mouse model	tripchlorolide; improves behavioral performance, protects dopaminergic neurons and inhibits astroglial responses	Hong et al. (2007)
		*in vitro*; preformed fibrils of human wild-type α-synuclein-induced mouse primary microglia	inhibits microglial activation by suppressing NF-κB activity *via* targeting the miR155–5p/SHIP1 pathway	Feng et al. (2019)
		*in vivo* and *vitro*; LPS-induced PD model	inhibits microglial activation by upregulating metabotropic glutamate receptor 5	Huang et al. (2018)
		*in vitro*; MN9D cell line	enhances autophagy in neuronal cells, promoting the clearance of α-synuclein	Hu et al. (2017)
	Celastrol	*in vivo*; *Drosophila* DJ-1A PD model	inhibits the reduction of dopaminergic neurons and increases brain dopamine content	Faust et al. (2009)
		*in vivo*; MPTP-induced mouse PD model	attenuates the loss of dopaminergic neurons, increases Hsp70 within dopaminergic neurons, and decreases the levels of NF-kB and TNF-α	Cleren et al. (2005)
		*in vivo*; MPTP-induced mouse PD model	exerts neuroprotective effects by promoting mitophagy	Lin et al. (2019)
		*in vivo*; AAV-mediated human α-synuclein overexpression PD model and the MPTP-induced PD mouse model	improve motor deficits by modulating the Nrf2-NLRP3-caspase-1 pathway	Zhang et al. (2021)
		*in vivo* and *vitro*; lactacystin-induced Wistar rats, SH-SY5Y cells and mouse primary cortical neurons	no neuroprotective effects	Konieczny et al. (2014)
		*in vitro*; rotenone-induced SH-SY5Y PD model	exerts neuroprotection by inducing autophagy, preserving mitochondrial function and inhibiting p38 MAPK	Deng et al., 2013; Choi et al., 2014
		*in vitro*; dendritic cells	mediates antigen trafficking in DCs, thus attenuating α-synuclein-specific T cell responses	Ng et al. (2022)
Multiple sclerosis	Triptolide	*in vivo*; C57 BL/6 mouse EAE model	delays the onset of EAE, attenuates the degree of inflammation and demyelination, improves behavirol deficits, and inhibits NF-kB-DNA binding activity	Wang et al. (2008)
		*in vivo*; C57 BL/6 mouse EAE model	LLDT-8; suppresses the severity of EAE by inhibiting T-cell activation	Fu et al. (2006)
		*in vivo*; SJL/J mouse EAE model	increases expression levels of Hsp70 and stabilisation of the NF-kB/IkBα complex	Kizelsztein et al. (2009)
		*in vivo*; cuprizone-induced toxic model	improves behavioral deficits and attenuates neuroinflammation by inhibiting NF-kB activation and promoting intrinsic myelin repair	Sanadgol et al. (2018)
	Celastrol	*in vivo*; relapsing-remitting EAE rat model	inhibits relapses and reduces clinical scores by modulating the Th1/Th2 cytokines profile (increases IL-10 expression but reduces TNF-α expression), inhibiting NF-κB and TLR2 expression, and reducing CD3^+^ T lymphocytic count	O'Brien et al., 2001; Abdin and Hasby, (2014)
		*in vivo*; EAE mouse model	exerts neuroprotective effects by inhibiting Th17 cell responses and attenuating cytokine production	Wang et al. (2015)
		*in vivo*; EAE mouse model	affects T-cell responses through the MAPK pathway, inhibiting SGK1 expression and incresing BDNF expression	Venkatesha and Moudgil, (2019)
		*in vivo*; EAE rat model	inhibiting the expression of iNOS and NF-kB and attenuating MS and optic neuritis	Yang et al. (2017)
Huntington’s diseases	Celastrol	*in vivo*; 3-nitropropionic acid-induced HD rat models	decreases striatal lesion voulme, increases the expression of Hsp70 in the striata, and attenuates astrogliosis	Cleren et al. (2005)
		*in vitro*; cell lines expressing mutant	inhibits polyglutamine aggregation by inducing HSF1 and increasing the expression of Hsp70	Zhang and Sarge, (2007)
		polyglutamine protein		
		*in vitro*; HdhQ111/Q111 knock-in mouse-derived striatal cell line	inhibits mutant huntingtin aggregation, and reverses the abnormal cellular localization of full-length mutant huntingtin	Wang et al. (2005)
Amyotrophic lateral sclerosis	Celastrol	*in vivo*; G93A SOD1 transgenic ALS mouse model	delays disease onset, improves motor deficits, increases the number of neurons, promotes Hsp70 expression, and reduces TNF-α and iNOS levels	Kiaei et al. (2005)
		*in vitro*; staurosporin or H2O2-induced primary motoneurons	activates the heat shock response (i.e. increases Hsp70 expression)	Kalmar and Greensmith, (2009)
		*in vitro*; H2O2-treated G93A SOD1 transfected NSC34 cells	reduces cell death by activating MEK/ERK and PI3K/AKT signaling pathways	Li et al. (2017)
Cerebral ischemia	Triptolide	*in vivo*; focal cerebral ischemia reperfusion rat model	improves neural function, attenuates neuronal apoptosis, and suppresses infiltration of neutrophils	Wei et al. (2004)
		*in vivo*; focal cerebral ischemia reperfusion rat model	exerts neuroprotection by inhibiting NF-kB activity	Jin et al., 2015; Bai et al., 2016a; Bai et al., 2016b
		*in vivo*; focal cerebral ischemia reperfusion rat model	exerts neuroprotective effects by inhibiting NF-kB/PUMA signaling pathway	Zhang et al. (2016)
		*in vivo* and *vitro*; focal cerebral ischemia reperfusion rat model, and OGD and TNF-α-stimulated SH-SY5Y cells	inhibits NF-kB and p38 MAPK signaling pathways, exerting neuroprotection	Hao et al. (2015)
		*in vivo*; focal cerebral ischemia reperfusion rat model	upregulates autophagy and downregulates apoptosis	Yang et al. (2015)
		*in vivo*; focal cerebral ischemia reperfusion rat model	downregulating apoptosis by activating the PI3K/AKT/mTOR signaling pathway	Li et al. (2015)
		*in vivo*; focal cerebral ischemic mouse model	improves cerebral ischemia by triggering BDNF-AKT signaling pathway and autophagy	Du et al. (2020)
		*in vivo*; focal cerebral ischemia reperfusion rat model	improves neurobehavioral scores, reduces brain damage, reduces levels of malondialdehyde and ROS, increases superoxide dismutase level, involving inhibition of Wnt/β-catenin signaling pathway	Pan and Xu, (2020)
		*in vivo* and *vitro*; chronic cerebral hypoperfusion mouse model, and OGD-stimulated primary oligodendrocytes and BV2 cells	alleviates white matter injury, protects against oligodendrocyte apoptosis directly, and inhibits microglial inflammation indirectly, involving increase of phosphorylation of the Src/AKT/GSK 3β singnaling pathway	Wan et al. (2022)
	Celastrol	*in vivo*; permanent middle cerebral artery occlusion mouse model	improves neurological function and reduces infarct volume in by attenuating the expression of NF-kB, p-c-Jun, and p-JNK	Li et al. (2012)
		*in vivo*; transient global cerebral ischemia reperfusion rat model	exerts neuroprotection, inhibits the expression of pro-inflammatory cytokines and MDA and elevates the levels of GSH and SOD, which is mediated by inhibiting HMGB1/NF-kB signaling pathway	Zhang et al. (2020)
		*in vivo* and *vitro*; focal cerebral ischemia reperfusion rat model and OGD-stimulated primary rat cortical neuron	directly binds to HMGB1, thus inhibiting the binding of HMGB1 to its downstream inflammatory components; inhibits NF-kB activity	Liu M et al. (2021)
		*in vivo* and *vitro*; permanent focal ischemia rat model and OGD-stimulated rimary neurons and microglia	exerts neuroprotective effects through an IL-33/ST2 axis-mediated M2 microglia/macrophage polarization	Jiang et al. (2018)
		*in vivo*; focal cerebral ischemia reperfusion mouse model	attenuates glycolysis and exerts neuroprotection by inhibiting HIF-1α/PDK1	Chen et al. (2022)
		*in vivo* and *vitro*; cerebral ischemia reperfusion mouse model and OGD-stimulated HT-22 cells	inhibits AK005401/MAP3K12 and activates PI3K/AKT signaling pathway, thus exerting neuroprotective effects	Wang et al. (2021)
Traumatic brain injury	Triptolide	*in vivo*; TBI rat model	improves neurological deficits and attenuates contusion volume, edema, cell apoptosis, decreases expressions of pro-inflammatory cytokines while increases level of anti-inflammatory cytokines	Lee et al. (2012)
	Celastrol	*in vivo*; TBI mouse model	improves neurobehavioral functions and protects neuronal cells by inducing Hsp70/Hsp110 expression	Eroglu et al. (2014)
Spinal cord injury	Triptolide	*in vivo* and *vitro*; SCI rat model and LPS-stimulated primary astrocytes	promotes spinal cord repair, inhibits inflammation, and attenuates astrogliosis and glial scar by inhibiting the JAK2/STAT3 pathway	Su et al. (2010)
		*in vivo*; SCI rat model	exerts neuroprotection by targeting the miR-96/IKKβ/NF-κB pathway and thus inhibiting microglial activation	Huang et al. (2019)
		*in vivo*; SCI mouse model	enhances autophagy and inhibits MAPK/ERK1/2 signaling pathway	Zhu et al. (2020)
	Celastrol	*in vitro*; SCI spinal cords model	reduces motorneuron death by inducing Hsp70 expression, while exerts limited protection on the lumbar motor network	Petrović et al. (2019)
		*in vivo* and *vitro*; SCI rat model and LPS + ATP-induced BV2 cells	attenuates microglial activation in the spinal cord, inhibits the expression of NF-kB, thus inhibiting the expression of NLRP3, caspase-1, GSDMD and inflammatory cytokines, while increases the levels of anti-inflammatory cytokines	Dai et al. (2019)
Epilepsy	Triptolide	*in vivo*; kainic acid-induced epilepsy rat model	protects neurons, which is associated with increased expression of neuron kv1.1 in the CA3 region of the hippocampus	Pan et al. (2012)
		*in vitro*; kainic acid-stimulated BV2 microglia	inhibits microglial activation, decreases MHC II expression in microglia by inhibiting AP-1/class II transactivator, which is related to neuronal death	Sun et al. (2018)
	Celastrol	*in vivo*; multiple-hit rat model	has a therapeutic effect by inhibiting NF-kB	Schiavone et al. (2021)
		*in vivo* and *vitro*; kainic acid-induced rats and hippocampal slices	inhibits NOX activation and rapid H_2_O_2_ release, thus alleviating epileptic seizure	Malkov et al. (2019)
		*in vivo*; mouse amygdala-kindling model	increases microglial activation in hippocampal CA1 and CA3 regions and reduces postkindling seizure thresholds	von Rüden et al. (2019)

In this review, we review the progress of research focusing on the therapeutic potential of triptolide and celastrol in neurological diseases, possible mechanisms of action of them, their toxicity and derivatives, and nanotechnology-based carrier system. This will contribute to the further understanding and in-depth studies of the therapeutic potential of these two components.

## 2 Effects of triptolide and celastrol on neurodegenerative diseases

### 2.1 Alzheimer’s disease

Alzheimer’s disease (AD) is a common neurodegenerative disease that can lead to symptoms such as dementia and affect people worldwide (Andrieu et al., 2015). The main pathological features of AD include the formation of extracellular pathological plaques (formed by the accumulation of amyloid-beta (Aβ) peptides) and the presence of intracellular neurofibril entanglements (formed by aggregations of hyperphosphorylated Tau proteins) (Montine et al., 2012). The etiology of AD is not yet fully understood, and a growing body of research suggests an important role for neuroinflammation in the pathology of AD (Dá Mesquita et al., 2016; Wang et al., 2017; Jiang et al., 2022). For example, studies have found that impairment of synaptic function in AD is associated with increased neuroinflammation (Dá Mesquita et al., 2016). Considering the anti-inflammatory and antioxidant effects of TWHF, many studies have reported the therapeutic effects of bioactive components of TWHF on AD in recent years.

#### 2.1.1 Triptolide

Triptolide is one of the important bioactive components of TWHF and it exerts neuroprotective and neurotrophic effects in AD (Li and Hao, 2019). Moreover, it has a small molecular weight and lipophilicity, which makes it have a better performance in the treatment of AD (Shui et al., 2010). The neuroprotective effects of triptolide on AD have been reported in both *in vivo* and *in vitro* studies. Wan et al. found that triptolide (intraperitoneal injection) could attenuate the degeneration of dendritic spines in hippocampal neurons in AD rats injected with Aβ 1-40 (Wan et al., 2014). Beside that, a study found that in the amyloid precursor protein/presenilin 1 (APP/PS1) double transgenic AD murine model, peripherally administered triptolide improved cognitive function, accompanied by reduced neuroinflammation and Aβ deposition, and that reduction of Aβ deposition may be associated with enhanced Aβ degradation (Cheng et al., 2014). In another study, in the APP/PS1 model of AD, intraperitoneally injected triptolide was found to reduce astrocyte proliferation and microglial activation in the hippocampal region, which was also related to the formation of pathological plaques and neurofibrillary tangles (Li et al., 2016; Villegas-Llerena et al., 2016). Furthermore, studies have revealed that intraperitoneal injection with tirptolide improved spatial memory deficits in APP/PS1 models of AD, involving inhibition of beta-site amyloid precursor protein cleaving enzyme 1 (BACE1) (Wang et al., 2014), and inhibition of inflammatory responses and mitogen-activated protein kinase (MAPK)s activity (Cui et al., 2016). In addition, Bakshi et al. reported that knockout of C-X-C chemokine receptor type 2 (CXCR2) inhibited Aβ production in the APP/PS1 model of AD, further elucidating the mechanism of Aβ production in AD (Bakshi et al., 2011). In *vitro* experiments revealed that in SH-SY5Y cells, triptolide could exert neuroprotective effects by inhibiting CXCR2 activity and reducing Aβ production (Wang et al., 2013b). These suggest that CXCR2 may play an important role in Aβ production. A variety of cells are involved in the development of AD, of which microglia, as important cells in the neuroinflammatory process, were reported in previous studies. Jiao et al. found that in Aβ 1-42-treated cultured rat microglia, triptolide inhibited tumor necrosis factor-α (TNF-α) and interleukin-1β (IL-1β) expression levels and thus exerted neuroprotective effects (Jiao et al., 2008). Furthermore, triptolide was reported to exert neuroprotective effects in neuronal PC12 cells. A previous study suggested that triptolide decreased the level of reactive oxygen species (ROS), thus attenuating apoptosis in PC12 cells stimulated by glutamate (He et al., 2003). In consistence with this, Gu et al. also found the attenuation of triptolide on apoptosis of PC12 cells (Gu et al., 2004). Beside that, Xu et al. demonstrated the neuroprotective effects of triptolide in Aβ 25-35-treated differentiated PC12 cells, which involves autophagy pathway and inhibition of oxidative stress (Xu et al., 2015; Xu et al., 2016). In view of the clinical use of triptolide, a recent study used nanoparticles loaded with triptolide to treat Aβ 1-42-treated differentiated PC12 cells and showed that this treatment reduced oxidative stress and consequently significantly inhibited cytotoxicity (Jia et al., 2021). The above experimental studies have shown that triptolide exerts neuroprotective effects in AD, which provides a theoretical basis for its clinical application.

In addition to neuroprotective effects, the neurotrophic effects of triptolide in AD has also been studied. It was found that primary astrocytes from rats treated with triptolide showed increased synthesis and release of nerve growth factor (NGF), a key neurotrophic factor that is essential for nerve cells (Xue et al., 2007). Moreover, in a cellular model of AD, treatment with triptolide increased the expression of synaptophysin in hippocampal neurons (Nie et al., 2012). Synaptophysin is an important protein of synaptic vesicle membrane and is mainly expressed at the presynaptic endings of the nervous system (García-Mesa et al., 2022). Recently, a study reported that intraperitoneally injected triptolide increased hippocampal neuroligin-1 (a cell adhesion protein, located on the excitatory postsynaptic membrane) expression in the APP/PS1 model of AD through epigenetic mechanisms (Lu et al., 2019). These studies demonstrate the neurotrophic effects of triptolide on AD models.

Above data suggest that triptolide exerts therapeutic effects on AD by the following mechanisms: 1) inhibiting Aβ deposition by inhibiting BACE1 or CXCR2 in neurons, 2) inhibiting MAPKs activity and attenuating neuroinflammation in microglia, 3) decreasing level of ROS and inhibiting oxidative stress in neurons, 4) attenuating apoptosis and stabilizing mitochondrial membrane potential in neurons, 5) enhancing autophagy in neurons, 6) exerting neurotrophic effects by promoting expression of NGF (in astrocytes), synaptophysin (in neurons), and neuroligin-1.

#### 2.1.2 Celastrol

Celastrol, one of the important bioactive components extracted from TWHF, has been the subject of many studies for the treatment of AD. Allison et al. reported that lipopolysaccharide (LPS) rat model intraperitoneally administered with celastrol exhibited improved performance in memory and learning activity tests (Allison et al., 2001). Moreover, subcutaneous treatment with celastrol was found to attenuate the accumulation of pathological plaque and microglial activation in the double transgenic Tg PS1/APPsw (human APP695sw mutation and the presenilin-1 mutation M146L) AD mice (Paris et al., 2010). In addition, the diabetes mellitus rat model showed reduced cognitive function and increased amyloid substance, similar to the alterations shown in the AD model, and celastrol treatment (intraperitoneal injection) inhibited these alterations (Liao et al., 2018). Recently, a study found that intraperitoneally administered celastrol improved learning and memory deficits in Aβ 25-35-microinjected rats (Xiao et al., 2021). Further, this study suggests that the neuroprotective effect of celastrol is associated with inhibition of nuclear factor-kappaB (NF-kB) activity, improved synaptic function and increased glucose metabolism. A recent study proposed that celastrol mixed in regular feed could reduce the accumulation of neurofibrillary tangles in the brains of two common animal models of AD (P301S microtubule associated protein tau mice and 3XTg mice) by activating transcription factor EB-mediated autophagy and lysosome biogenesis, thereby attenuating memory deficits in AD mice. Similarly, these effects of celastrol were also found in N2a cells (Yang et al., 2022b).

Beside that, other related *in vitro* experiments have further explored the possible mechanisms involved in the neuroprotective effects of celastrol on AD. Low doses of celastrol have been reported to inhibit the production of TNF-α and IL-1β by human monocytes and macrophages, the expression of major histocompatibility complex II (MHC II) molecules of microglia, and the production of inducible nitric oxide synthase (iNOS) in endothelial cells (Allison et al., 2001). It is well known that NF-kB is an important regulator in inflammatory responses. Paris et al. found that celastrol could attenuate NF-kB activity in HEK293 cells; moreover, they also demonstrated the inhibition of celastrol on Aβ production in 7 W CHO cells overexpressing wild-type human APP, which is related to the inhibition of BACE-1 (β-site APP cleaving enzyme) (Paris et al., 2010). BACE-1 is a major β-secretase, exerting effects on proteolysis of the β-APP to obtain Aβ peptides (Vassar et al., 1999). Furthermore, using molecular docking and *in vitro* assays, a study found that celastrol exerts neuroprotective effects in IMR-32 cells by inhibiting inhibitor κB kinase (IKK), which is the converging point in the activation of NF-κB (Veerappan et al., 2017). Moreover, it was reported that celastrol inhibited the production of Aβ in LPS-treated H4-APP cells, attenuated NF-kB activity and suppressed cyclooxygenase-2 (COX-2) expression. In addition, the effects of celastrol on increased levels of heat shock protein (Hsp) 70 and Bcl-2 expression have been reported (Zhao et al., 2014). Heat shock proteins are a series of proteins that have effects on folding of proteins. Among them, Hsp27 and Hsp70 are thought to have neuroprotective effects and their low levels are associated with AD (Chow et al., 2014). It was found that celastrol induced the increase in the expression of Hsp27 and Hsp70 in mature brain cortical cultures (Chow et al., 2013). However, in another study, celastrol was found to have no effect on the expression of Hsp70 in Aβ 1-42-treated SH-SY5Y cells, but could inhibit the expression of Hsp90 (Cao et al., 2018). This suggests that celastrol can exert neuroprotective effects through different pathways.

These studies reveal the mechanisms of therpaeutic effects of celastrol on AD: 1) inhibiting NF-kB activity and attenuating neuroinflammation in microglia and neurons, 2) reducing accumulation of neurofibrillary tangles by activating transcription factor EB-mediated autophagy and lysosome biogenesis, 3) inhibiting Aβ production by inhibition of BACE-1 in neurons, 4) increasing the expression of Hsp70 in mature brain cortical cultures while decreasing the expression of Hsp90 in neurons.

### 2.2 Parkinson’s disease

Parkinson’s disease (PD), a movement disorder, is the second most common neurodegenerative disease worldwide, mainly affecting older people (Kalia and Lang, 2016). Its main pathological feature is the progressive loss of dopaminergic neurons in the substantia nigra (Dauer and Przedborski, 2003). The main clinical manifestations of PD are resting tremor, bradykinesia, rigidity and postural abnormalities (Postuma et al., 2015). In addition, non-motor symptoms such as autonomic abnormalities, olfactory impairment and cognitive deficits also play an important role in the diagnosis of PD (Martinez-Martin et al., 2011). The pathogenesis of PD is not yet fully understood. Studies have shown that α-synuclein transmission is important in the pathogenesis of PD, which has made α-synuclein a hot topic in the field of PD research in recent years (Kurowska et al., 2011; Ganguly et al., 2022). Moreover, research on neuroinflammation in the pathogenesis of PD has also received widespread attention (Jenner and Olanow, 2006; Arena et al., 2022). As mentioned earlier, TWHF has anti-inflammatory properties. In recent years, there are many studies on the therapeutic effects of its extracts on PD.

#### 2.2.1 Triptolide

Triptolide, as one of the main bioactive components of TWHF exerting anti-inflammatory effects, has been the subject of many *in vivo* and *in vitro* studies for its therapeutic effects on PD. Zhou et al. developed an inflammatory rat model by LPS intranigral injection and investigated the therapeutic effects of triptolide by intraperitoneal treatment with triptolide for 24 consecutive days. The results showed that triptolide treatment protected dopaminergic neurons and reduced the expression of pro-inflammatory cytokines, such as TNF-α and IL-1β, thereby exerting neuroprotective effects (Zhou et al., 2005). In line with this, in another inflammatory rat model using LPS induction, triptolide treatment protected dopaminergic neurons and inhibited microglial activation (Li G. et al., 2006). Moreover, a study investigated the neuroprotective effects of triptolide in hemiparkinsonian rats using a 1-methyl-4-phenyl pyridinium (MPP+)-induced rat model. They found that intraperitoneally injected triptolide improved behavioral performance in this model and which may be associated with protection of dopaminergic neurons and inhibition of microglial activation (Gao et al., 2008).

Similar results have been reported in *vitro* experiments. In primary mesencephalic neuron/glia mixed culture, Li et al. reported the inhibition of triptolide on the decrease in [3H]dopamine uptake and loss of tyrosine hydroxylase-immunoreactive neurons induced by LPS, and underlying mechanism may be related to the inhibition of microglial activation (Li et al., 2004). In this study, they also found that treatment with triptolide inhibited the overproduction of TNF-α and NO. In addition, a study also found that triptolide inhibited TNF-α and NO production in primary microglial cultures (Zhou et al., 2003). Furthermore, a research used a novel extraction method to obtain extracts from TWHF and found that the extracts protected dopaminergic neurons in rat mesencephalic neuron-glia cultures induced by LPS, inhibited microglial activation and TNF-α and NO expression (Liu et al., 2010). Most of the triptolide in the extracts obtained by this new extraction method was converted to tripchlorolide, an analogue of triptolide (Li B. et al., 2006). The therapeutic effects of tripchlorolide on PD are currently being studied *in vitro* and *in vivo*. For example, tripchlorolide was found to exert neuroprotective and neurotrophic effects on dopaminergic neurons by promoting axonal elongation and protecting dopaminergic neurons, as well as increasing *brain-derived neurotrophic factor (BDNF)* mRNA expression (Li et al., 2003). In another study, tripchlorolide was reported to protect dopaminergic neurons, inhibit the overproduction of TNF-α and IL-2, and thus exerting neuroprotective effects in rat models of PD (Cheng et al., 2002). Beside that, In a mouse model of 1-methyl-4-phenyl-1,2,3,6-tetrahydropyridine (MPTP)-induced PD, intraperitoneally treated with tripchlorolide improved behavioral performance, protected dopaminergic neurons and inhibited astroglial responses, thereby exerting neuroprotective effects (Hong et al., 2007). Recently, it has been shown that the inhibitory effect of triptolide on microglial activation during PD treatment may be achieved by targeting the microRNA155–5p/SHIP1 pathway or by upregulating metabotropic glutamate receptor 5 (intraperitoneal injection) (Huang et al., 2018; Feng et al., 2019). In addition, enhanced autophagy in neurons, which in turn promotes the clearance of α-synuclein, is one of the possible mechanisms of triptolide to treat PD (Hu et al., 2017).

The above studies illustrate the therapeutic effects of triptolide and tripchlorolide on PD models, which is associated with the following mechanisms: 1) attenuating neuroninflammation in microglia and neurons, 2) promoting the clearance of α-synuclein in neurons by enhancing autophagy, 3) exerting neurotrophic effects by incresing the expression of *BDNF* mRNA in neurons. These suggest that triptolide and tripchlorolide may be potential candidates for clinical treatment of PD.

#### 2.2.2 Celastrol

Considering the anti-inflammatory effects of celastrol, there are many studies focusing on its therapeutic effects on PD. Faust et al. reported that in the *Drosophila* DJ-1A model of PD, celastrol inhibited the reduction of dopaminergic neurons and increased brain dopamine content, thereby exerting neuroprotective effects in the PD model (Faust et al., 2009). Beside that, the therapeutic effects of celastrol have also been reported in mouse models of MPTP-induced PD. A previous study reported that intraperitoneally treated with celastrol attenuated the loss of dopaminergic neurons, increased Hsp70 within dopaminergic neurons, and decreased the levels of NF-kB and TNF-α (Cleren et al., 2005). Furthermore, a recent study suggested that the neuroprotective effects of celastrol (intraperitoneal injection) on MPTP-induced PD mouse models was associated with activation of mitophagy (Lin et al., 2019). However, Zhang et al. reported some contradictory results when they found that celastrol inhibited PTEN-induced kinase 1 (PINK1)-dependent mitophagy (Zhang et al., 2019). This may be due to the complicated mechanisms of action of celastrol. Moreover, the therapeutic mechanisms of celastrol has been further explored. Using the adeno-associated virus (AAV)-mediated human α-synuclein overexpression PD model and the MPTP-induced PD mouse model, a team of researchers found that intraperitoneally injected with celastrol can improve motor deficits and thus exert neuroprotective effects by modulating the nuclear factor-erythroid 2-related factor 2 (Nrf2)- Nod-like receptor family, pyrin-domain-containing 3 (NLRP3)-caspase-1 pathway (Zhang et al., 2021). However, a study found that celastrol (intraperitoneal treatment) did not exert neuroprotective effects in Wistar rats in the presence of lactacystin-induced ubiquitin-proteasome system (UPS) inhibition (Konieczny et al., 2014). Consistent with this, celastrol also did not exert neuroprotective effects in SH-SY5Y cells and mouse primary cortical neurons, which incubated with lactacystin. Moreover, the therapeutic effects of celastrol can be found in the rotenone-induced SH-SY5Y model of PD. For example, Deng et al. reported the protection of celastrol on rotenone-induced SH-SY5Y, involving the induction of autophagy (Deng et al., 2013). Further study suggests that this protective effect may also involve preservation of mitochondrial function and inhibition of p38 MAPK (Choi et al., 2014). Recently, a study focused on the role of celastrol on dendritic cells (DCs), and the results demonstrated that celastrol could mediate antigen trafficking in DCs, thus attenuating α-synuclein-specific T cell responses (Ng et al., 2022), and this may be beneficial to the treatment of PD.

These data suggest that mechanisms of therapeutic effects of celastrol on PD: 1) increasing the expression of Hsp70 in neurons, 2) attenuating neuroinflammation, 3) activating mitophagy, 4) inducing autophagy in neurons, 5) preserving mitochondrial function and inhibiting p38 MAPK, 6) mediating antigen trafficking in DCs, thus attenuating α-synuclein-specific T cell responses.

### 2.3 Multiple sclerosis

Multiple sclerosis (MS) is a demyelinating, neurodegenerative disease that occurs mainly in women. Its main pathological features are early demyelination and late neurodegeneration (Dobson and Giovannoni, 2019). The pathogenesis of MS is not well understood, while it is generally regarded as an autoimmune disease in which autoimmunity and neuroinflammation play a critical role (Segal, 2019). There is currently no cure for MS, and many studies have investigated the therapeutic effects of extracts of TWHF, which has anti-inflammatory properties, on it.

#### 2.3.1 Triptolide

To date, there is no cellular model that simulates the onset of MS well; therefore, most basic research on MS has been conducted using animal models. Among the many animal models, the experimental autoimmune encephalomyelitis (EAE) is the most widely used animal model that simulates the onset of MS (Gehrmann et al., 1993). Several studies have shown that triptolide and its analogues can exert neuroprotective effects against EAE. For example, a previous study reported the neuroprotective effects of triptolide on EAE models of C57 BL/6 mice, and results showed that intraperitoneally treated with triptolide could delay the onset of EAE, attenuate the degree of inflammation and demyelination, and improve behavirol deficits; moreover, the inhibition of NF-kB-DNA binding activity was also found (Wang et al., 2008). In addition, Fu et al. found that intraperitoneally injected with (5R)-5-hydroxytriptolide (LLDT-8), one of the derivatives of triptolide, could suppress the severity of EAE models of C57 BL/6 mice by inhibiting T-cell activation (Fu et al., 2006). The therapeutic effects of triptolide (oral administration) was also found in the EAE model of SJL/J mice, involving increased expression levels of Hsp70 and stabilisation of the NF-kB/inhibitor-kappa-B alpha (IkBα) complex (Kizelsztein et al., 2009). In addition to the EAE model, there is also a cuprizone induced toxic model that can be used to study MS, which is primarily used to study damage to oligodendrocytes and myelin sheaths during MS (Abakumova et al., 2015). A recent study demonstrated that, in cuprizone induced toxic models, triptolide administered intraperitoneally improved behavioral deficits and attenuated neuroinflammation by inhibiting NF-kB activation and promoting intrinsic myelin repair (Sanadgol et al., 2018). Together, these related studies demonstrate that inhibiting NF-kB activity and attenuating neuroinflammation are crucial in therapeutic effects of triptolide on MS.

#### 2.3.2 Celastrol

There are also basic studies on the therapeutic effects of celastrol on MS. It was shown that in a relapsing-remitting EAE rat model, celastrol (intraperitoneal injection) inhibited relapses and reduced clinical scores by modulating the Th1/Th2 cytokines profile (increased IL-10 expression while reduced TNF-α expression), inhibiting NF-κB and Toll-like receptor 2 (TLR2) expression, and reducing CD3^+^ T lymphocytic count (O'Brien et al., 2001; Abdin and Hasby, 2014). MS is a classic autoimmune disease in which the imbalance between Th17 and Treg cells is important in the pathophysiology, as evidenced by the promotion of pathological Th17 responses, and the inhibition of Treg responses (Holmøy, 2008; Di Mitri et al., 2015). Therefore, several studies have further investigated the effects of celastrol on T cells in EAE. For example, Wang et al. reported that intraperitoneally treated with celastrol exerted neuroprotective effects on EAE by inhibiting Th17 cell responses and attenuating cytokine production in bone-marrow derived dendritic cells (Wang et al., 2015). Moreover, another study suggested that the effect of celastrol (intraperitoneal treatment) on T-cell responses is mediated through the MAPK pathway, in which serum/glucocorticoid regulated kinase 1 (SGK1) expression is suppressed, yet BDNF expression is increased (Venkatesha and Moudgil, 2019). In addition, a study using an EAE rat model found that intraperitoneally injected with celastrol inhibited the expression of iNOS and NF-kB and attenuated MS and optic neuritis in the models (Yang et al., 2017). Similar to triptolide, NF-kB is also a key signaling pathway in therapeutic effects of celastrol on MS. Besides that, celastrol can modulate Th1/Th2 cytokines profile and inhibit Th17 cell responses through the MAPK pathway, thus attenuating neuroinflammation.

### 2.4 Huntington’s diseases and amyotrophic lateral sclerosis

Huntington’s diseases (HD) is a fatal, progressive neurodegenerative disease. It is caused by excesively expanded CAG repeats in the gene and is an autosomal dominant disorder (Rai et al., 2019). Clinical manifestations of HD include motor dysfunction, cognitive impairment and psychiatric disturbances (Ross and Tabrizi, 2011). Currently, HD cannot be completely cured. Some studies have found that celastrol has therapeutic effects on HD, but there are no reports of triptolide in HD. Cleren et al. reported the neuroprotection of celastrol (intraperitoneal injection) on 3-nitropropionic acid-induced HD rat models, and they found that celastrol could decrease striatal lesion voulme, increase the expression of Hsp70 in the striata, and attenuate astrogliosis (Cleren et al., 2005). Considering the neuroprotective effects of heat shock proteins and the previously mentioned relationship between celastrol and heat shock proteins, another study explored the therapeutic effects of celastrol and its effect on the heat shock response in cellular models of HD. The results showed that celastrol could inhibit polyglutamine aggregation by inducing heat shock factor 1 (HSF1) and then increasing the expression of Hsp70 (Zhang and Sarge, 2007). In addition, a study used a HdhQ111/Q111 knock-in mouse-derived striatal cell line to investigate the effects of celastrol and found that celastrol inhibited mutant huntingtin aggregation, and reversed the abnormal cellular localization of full-length mutant huntingtin (Wang et al., 2005).

Amyotrophic lateral sclerosis (ALS), a neurodegenerative disease, is an common adult motor neuron disease that can lead to progressive muscle atrophy and even death. Although the pathogenesis of ALS has not been fully elucidated, many studies have shown that the genetic component plays an important role in its development. Current treatment for ALS is mainly symptomatic, while there are some basic studies showing the therapeutic effects of celastrol in ALS models. As with HD, the therapeutic effect of triptolide in ALS has not been reported. A study using celastrol in a G93A-superoxide dismutase 1 (SOD1) transgenic mouse model of ALS found that orally administrated celastrol delayed disease onset, improved motor deficits, and increased the number of neurons, promoted Hsp70 expression, and reduced TNF-α and iNOS levels in the spinal cord (Kiaei et al., 2005). In addition, celastrol was found in *vitro* experiments to exert neuroprotective effects in primary motoneurons induced by staurosporin or H_2_O_2_ by activating the heat shock response (i.e. increasing Hsp70 expression) (Kalmar and Greensmith, 2009). Similar neuroprotective effects were found in another *in vitro* study. Specifically, Li et al. used H_2_O_2_ to treat G93A-SOD1 transfected NSC34 cells as a cellular model of ALS and found that celastrol reduced cell death, involving activation of mitogen-activated protein kinase (MEK)/extracellular regulated protein kinases (ERK) and phosphoinositide 3-kinase (PI3K)/serine-threonine kinase (AKT) signaling pathways (Li et al., 2017).

In conclusion, the therapeutic effects of celastrol in HD and ALS are closely related to heat shock proteins, especially Hsp70, although other mechanisms such as inhibition of astrogliosis exist.

## 3 Effects of triptolide and celastrol on brain and spinal cord injury

### 3.1 Cerebral ischemia

With an ageing population, the incidence of stroke is increasing, placing a huge burden on society and families. Of these, ischemic stroke is the most common type of stroke and is the third leading cause of disability worldwide (Benjamin et al., 2018; Meng et al., 2021). The therapeutic time window for ischemic stroke is narrow and there are significant financial and care costs for the treatment and rehabilitation of patients with ischemic stroke, so it is important that patients suffering with ischemic stroke are treated promptly and effectively (Sun et al., 2020). Currently, the main treatment options for ischemic stroke episodes include chemical thrombolysis (intravenous) and mechanical thrombectomy (intra-arterial) (Moussaddy et al., 2018). However, these treatments lead to a classic pathophysiological process known as ischemia/reperfusion injury. During cerebral ischemia/reperfusion injury, multiple pathophysiological mechanisms are involved and interact with each other, including oxidative stress, inflammatory responses, neuronal apoptosis and autophagy (Meng et al., 2021). Therefore, many studies have used models of cerebral ischemia/reperfusion injury to conduct research related to cerebral ischemia.

#### 3.1.1 Triptolide

Middle cerebral artery occlusion (MCAO) is a common animal model of cerebral ischemia, and many studies have demonstrated the therapeutic effects of triptolide on MCAO. For example, Wei et al. found that triptolide improved neural function, attenuated neuronal apoptosis, and suppressed infiltration of neutrophils in rat with focal ischemia/reperfusion (Wei et al., 2004). Moreover, since inflammatory responses play an important role in cerebral ischemia/reperfusion injury, several studies further explored the effect of triptolide on NF-kB, an essential regulator of inflammatory responses, in MCAO, and reported the inhibitory effect of triptolide on NF-kB activity (Jin et al., 2015; Bai et al., 2016a; Bai et al., 2016b). In addition, Zhang et al. found that triptolide inhibited NF-kB/p53 upregulated modulator of apoptosis (PUMA) signaling pathway, which in turn exerts neuroprotective effects against MCAO (Zhang et al., 2016). This inhibition has also been reported in cellular models. A previous study, using MCAO rats and oxygen-glucose deprivation (OGD) SH-SY5Y cells, found that triptolide inhibited NF-kB and p38 MAPK signaling pathways to exert neuroprotection (Hao et al., 2015). In addition to its effects on NF-kB activity, there are several studies showing that triptolide exerts protective effects against cerebral ischemia by affecting autophagy. For example, Yang et al. found the upregulation of autophagy and downregulation of apoptosis in MCAO treated with triptolide (Yang et al., 2015). In a further study, the team found that the downregulation of apoptosis by triptolide in MCAO was through activation of the PI3K/AKT/mammalian target of rapamycin (mTOR) signaling pathway (Li et al., 2015). Beside that, neurobehavioral function in MCAO was improved by the combined use of the colony-stimulating factor 1 receptor inhibitor and triptolide, involving mechanisms including activation of autophagy and BDNF-AKT signaling pathways (Du et al., 2020). Pan et al. also reported the therapeutic effects of triptolide on MCAO. In their experimental results, triptolide could improve neurobehavioral scores, reduce brain damage, exert anti-inflammatory and antioxidant effects (reduce levels of malondialdehyde (MDA) and ROS, increase superoxide dismutase (SOD) level), and these effects were associated with inhibition of Wnt/β-catenin signaling pathway (Pan and Xu, 2020). In addition, right unilateral common carotid artery occlusion (rUCCAO), which simulates chronic cerebral hypoperfusion, can also be used for cerebral ischemia related research (Yoshizaki et al., 2008). A recent study using rUCCAO and OGD reported the therapeutic effects of triptolide *in vitro* and *in vivo*, including alleviating white matter injury, protecting against oligodendrocyte apoptosis directly, and inhibiting microglial inflammation indirectly, and the related mechanism was to increase the phosphorylation of the Src/AKT/GSK 3β singnaling pathway (Wan et al., 2022).

Take together, triptolide exerts neuroprotective effects on cerebral ischemia by the following mechanisms: 1) inhibiting NF-kB signaling pathway, involving inhibition of NF-kB/PUMA signaling pathway and inhibition of p38 MAPK/NF-kB signaling pathway, 2) enhancing autophagy, 3) inhibiting apoptosis by activating PI3K/AKT/mTOR signaling pathway, 4) upregulating BDNF-AKT signaling pathways, 5) exerting antioxidant effects by inhibiting Wnt/β-catenin signaling pathway, 6) inhibiting oligodendrocyte apoptosis by upregulating the phosphorylation of the Src/AKT/GSK 3β pathway.

#### 3.1.2 Celastrol

Many studies have also demonstrated the therapeutic effects of celastrol on cerebral ischemia and related mechanisms. To study the therapeutic effects of celastrol on cerebral ischemia, Li et al. used a permanent middle cerebral artery occlusion (pMCAO) model (Li et al., 2012). It is worth noting that the process of pMCAO does not involve reperfusion, that is, it does not belong to the ischemia/reperfusion injury model, while it is also an important model for cerebral ischemia research. Their results indicated that celastrol improved neurological function and reduced infarct volume in pMCAO possibly through suppressing the expression of NF-kB, p-c-Jun, and p-(c-Jun N-terminal kinase) JNK. In another study, the transient global cerebral ischemia reperfusion (tGCI/R) model was used, and the investigators obtained similar neuroprotective effects as in the previous pMCAO study. The investigators also found that celastrol could suppress the expression of pro-inflammatory cytokines and MDA and elevate the levels of glutathione (GSH) and SOD in tGCI/R. Moreover, these protective effects of celastrol could be mediated by inhibition of high mobility group protein 1 (HMGB1)/NF-kB signaling pathway (Zhang et al., 2020). Further studies investigated the association between celastrol and HMGB1/NF-kB. Liu et al. found that under conditions of MCAO or OGD, celastrol could directly bind to HMGB1 and in turn inhibit the binding of HMGB1 to its downstream inflammatory components; moreover, celastrol could also target NF-kB and inhibit its activity (Liu D. D. et al., 2021). As important immune cells, microglia/macrophages play an important role in regulating inflammatory response and are involved in the pathophysiology of cerebral ischemia (Hu et al., 2012). Studies have shown that microglia/macrophages have different phenotypes, with M1 microglia releasing neurotoxic substances that exacerbate brain damage, and M2 microglia exerting neuroprotective effects by producing neurotrophic substances and removing debris (Brifault et al., 2015). A study reported neuroprotective effects of celastrol in MCAO and further found in OGD that this neuroprotection was through an IL-33/ST2 axis-mediated M2 microglia/macrophage polarization (Jiang et al., 2018). In addition, glycolysis plays a role in the pathogenesis of cerebral ischemia, and some studies have found that inhibition of excessive glycolysis can protect neurons against OGD (Burmistrova et al., 2019; Vojinovic et al., 2020). Chen et al. reported that celastrol inhibited hypoxia inducible factor-1α (HIF-1α)/pyruvate dehydrogenasekinase1 (PDK1), thus attenuating glycolysis and exerting neuroprotection (Chen et al., 2022). Besides that, studies of long noncoding RNAs (LncRNAs) have received much attention in recent years. A recent study focused on the role of LncRNAs-AK005401 in the treatment of MCAO with celastrol, and showed that celastrol exerts neuroprotective effects through inhibition of AK005401/MAP3K12 and activation of PI3K/AKT (Wang et al., 2021). In another study, researchers analyzed the profiles of LncRNAs and mRNAs following celastrol treatment of MCAO by RNA-sequencing and bioinformatic analysis and experimentally verified the significantly different LncRNAs and mRNAs (Liu J. et al., 2022). Liu et al. used ultra-performance liquid chromatography-tandem mass spectrometry (UPLC/MS) to perform lipidomic analysis after celastrol treatment of MCAO and obtained some differentially expressed components (Liu M. et al., 2021). These studies have provided a theoretical basis for subsequent in-depth investigations.

Above studies suggest the mechanisms of therapeutic effects of celastrol on cerebral ischemia: a). attenuating neuroinflammation by inhibiting HMGB1/NF-kB signaling pathway, inhibiting the phosphorylation of the JNK, or promoting an IL-33/ST2 axis-mediated M2 microglia/macrophage polarization, b). suppressing glycolysis by inhibiting HIF-1α/PDK1, c). inhibiting AK005401/MAP3K12 and activating PI3K/AKT signaling pathway.

### 3.2 Traumatic brain injury and spinal cord injury

Traumatic brain injury (TBI) is a type of brain trauma that can lead to long-term disability and possibly secondary neurodegenerative disorders (Sivanandam and Thakur, 2012; Humphreys et al., 2013). The role of neuroinflammation in the pathophysiology of TBI cannot be ignored. Numerous studies have reported the damaging effects of neuroinflammation on brain cells in TBI (Kunz et al., 2002; Strauss, 2008). The two main bioactive components of TWHF, triptolide and celastrol, have been reported to have anti-inflammatory and therapeutic effects in models of TBI. For example, a study found that triptolide improved neurological deficits and attenuated contusion volume, edema, and cell apoptosis in TBI-induced brains. Moreover, it also reported the decreased expressions of pro-inflammatory cytokines while the increased level of anti-inflammatory cytokines (Lee et al., 2012). In addition, celastrol was also found to improve neurobehavioral functions and protect neurons in TBI, and the relevant mechanisms involved the induction of Hsp70/Hsp110 expression (Eroglu et al., 2014).

Spinal cord injury (SCI) is also a neurological disease that can lead to long-term disability, with a high mortality and varying clinical symptoms depending on the degree of lesions (Coyoy-Salgado et al., 2019). In terms of pathology, SCI can be divided into primary, secondary and chronic phases, with oxidative stress and inflammation playing an important role in the pathophysiology of it (Fehlings and Tator, 1995; McDonald and Sadowsky, 2002). Su et al. showed that triptolide could promote spinal cord repair in SCI by attenuating astrogliosis and inflammation, and that attenuation of astrogliosis is *via* inhibition of the Janus kinase 2 (JAK2)/signal transducer and activator of transcription 3 (STAT3) pathway (Su et al., 2010). In addition to the modulation of astrocytes, modulation of microglia has also been reported in the neuroprotective effects of triptolide in SCI. A study found that in SCI, the neuroprotection of celastrol on spinal cord injury was achieved by targeting the miR-96/IKKβ/NF-κB pathway and thus inhibiting microglial activation (Huang et al., 2019). Moreover, enhanced autophagy and inhibition of MAPK/ERK1/2 signaling pathway were also found to be involved in the promotion of spinal cord injury repair by triptolide in SCI (Zhu et al., 2020). Furthermore, the neuroprotection of celastrol on SCI was also reported. For example, an *in vitro* study found that celastrol significantly reduced motorneuron death. As mentioned above, celastrol plays an important role in the induction of Hsp70 in diseases, and there are similar results in the study of SCI, that is, celastrol can play a neuroprotective role by inducing the expression of Hsp70. However, the limited protection of celastrol on the lumbar motor network was found in this study, suggesting that the therapeutic effects of celastrol in SCI are limited (Petrović et al., 2019). In addition, Dai et al. investigated the effects of celastrol on pyroptosis in SCI (Dai et al., 2019). Pyroptosis is a form of cell death that is distinct from necrosis and apoptosis and is associated with immunity and inflammation (Shi et al., 2015). They investigated the effects and mechanisms of celastrol in SCI rats and LPS/adenosine triphosphate (ATP)-induced BV2 cells and found that celastrol attenuated the activation of microglia in the spinal cord, inhibited the expression of pyroptosis-related molecules (NLRP3, caspase-1 and gasdermin D (GSDMD)) and inflammatory cytokines, and increased the levels of anti-inflammatory cytokines. Beside that, their study found that celastrol inhibited the expression of NF-kB, one of the upstream promoters of pyroptosis (Li et al., 2021). These suggest that the protective effects of celastrol against SCI is achieved, at least in part, through the inhibition of pyroptosis. These data suggest that triptolide and celastrol exert therapeutic effects by the following mechanisms: a). attenuating neuroinflammation by targeting miR-96/IKKβ/NF-κB, NF-kB/NLRP3/caspase-1 or MAPK/ERK1/2 pathway, or inducing the expression of Hsp70, b). enhancing autophagy, c). attenuating astrogliosis *via* inhibiting JAK2/STAT3 pathway.

## 4 Effects of triptolide and celastrol on epilepsy

Epilepsy is a chronic neurological disease with various clinical manifestations, and its typical clinical manifestation is epileptic seizures. As a common brain disorder, epilepsy affects more than 70 million people worldwide, adversely affecting the quality of life of patients (Khan et al., 2020). The etiology of epilepsy is not fully understood. Studies have shown that multiple factors are involved in the pathogenesis of epilepsy, such as genes, infections, structures and immunity (Singh and Trevick, 2016). Abnormal neuronal discharge in the brain is thought to be an important cause of epileptic seizures in epilepsy patients. Such abnormal discharge is associated with loss of neurons, dysfunction of glial cells, and changes in neurotransmitters or neuromodulation. Further, recurrent abnormal discharge of neurons in the brain can lead to neuronal abnormalities and synaptic remodelling, exacerbating epilepsy (He et al., 2021). Currently, drugs such as carbamazepine, phenytoin sodium and valproic acid are commonly used to treat epilepsy. However, their use has some side effects, whereas natural medicines have fewer side effects and are safer. Therefore, research on natural medicines for epilepsy is of clinical importance. Triptolide and celastrol have been found to have potential therapeutic implications in the studies of epilepsy.

### 4.1 Triptolide

Kainic acid is often used to induce epileptic seizures. Pan et al. induced epileptic seizures in rats by intracarotid subcutaneous injection of kainic acid, and in the treated rats, triptolide (15 μg/kg) was administered intraperitoneally daily. The results showed that triptolide had a protective effect on neurons in epileptic rats, and this was associated with increased expression of neuron kv1.1 in the CA3 region of the hippocampus (Pan et al., 2012). Glial cells play an important role in the pathogenesis of epilepsy, among which microglia are innate immune cells in the central nervous system (CNS), and the expression of MHC II in microglia increases under inflammatory conditions, which can activate CD4^+^ T cells and promote the inflammatory response (Nakahara et al., 2010). In epileptic rats, this was related to neuronal death (Shaw et al., 1994). An *in vitro* study used kainic acid-stimulated BV2 microglia to investigate the effect of triptolide on microglia. They found that microglia treated with triptolide for 2 h resulted in inhibition of microglial activation (morphology). Furthermore, treatment with triptolide significantly inhibited MHC II expression in microglia, and further promoter predictions and experimental results showed that this inhibition correlated with the inhibition of AP-1/class II transactivator (Sun et al., 2018).

### 4.2 Celastrol

In addition, several studies have also reported on the therapeutic effects of celastrol on epilepsy. Study has reported that researchers used a multiple-hit rat model to explore the therapeutic effects of celastrol on infantile spasms. Considering the anti-inflammatory effects of celastrol and the important role of inflammation and immunity in epilepsy, their findings found that celatrol could have a therapeutic effect on the disease by inhibiting NF-kB (Schiavone et al., 2021). Moreover, a study found that in a disease model of epilepsy, N-methyl-d-aspartate receptor-mediated activation of nicotinamide adenine dinucleotide phosphate oxidase (NOX) induces rapid release of H_2_O_2_, which is associated with epileptic seizures. In hippocampal slices and kainic acid-induced rats, treatment with celatrol inhibited NOX activation and rapid H_2_O_2_ release, thereby alleviating epileptic seizures (Malkov et al., 2019). However, a study reported the conflicting results, demonstrating that celastrol increases activation of microglia in hippocampal CA1 and CA3 regions and reduces postkindling seizure thresholds (von Rüden et al., 2019). This makes the effects of celastrol on epilepsy even more complex and requires more experiments to explore.

In conclusion, the research on the therapeutic mechanisms of triptolide and celastrol in epilepsy is still not deep enough, and more in-depth studies are needed to further clarify their mechanisms.

## 5 Molecular mechanisms of triptolide and celastrol for therapeutic potential

**FIGURE 2:**
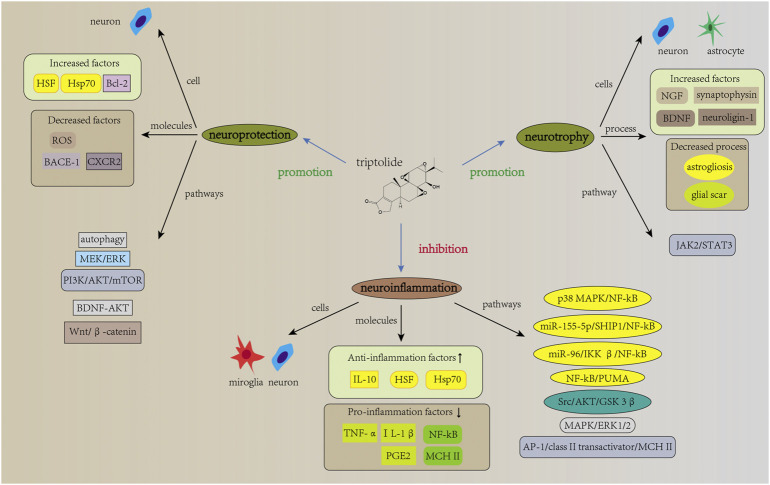
Mechanisms of action of triptolide in neurological diseases. The mechanisms of action of triptolide in neurological diseases involves neuroinflammation, neurotrophic effects, and other neuroprotective effects. It attenuates neuroinflammation and regulate inflammation levels in microglia and neuron, as evidenced by increased expression of anti-inflammatory factors and decreased expression of pro-inflammatory factors. Signaling pathways such as NF-kB are involved in this regulation. In addition, current studies have reported neurotrophic effects of triptolide for neurological diseases. Triptolide increases the expression levels of NGF, synaptophysin, neuroligin-1, and *BDNF* mRNA, and it also inhibits astrogliosis and excessive glial scar, which is associated with the regulation of JAK2/STAT3 signaling pathway. Furthermore, triptolide can act directly on neurons and thus exert neuroprotective effects by affecting other signaling pathways such as autophagy and apoptosis.

**FIGURE 3:**
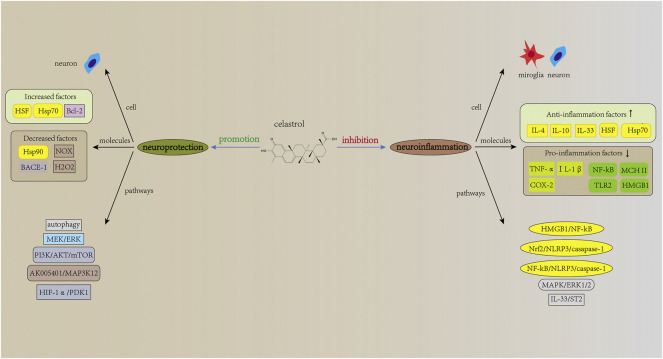
Mechanisms of action of celastrol in neurological diseases. Similar to triptolide, celastrol has therapeutic potential in neurological diseases, with related mechanisms of action including inhibition of neuroinflammation and promotion of neuroprotection. The NF-kB signaling pathway is also involved in the action of celastrol, and celastrol can also act directly on neurons, thereby exerting neuroprotective effects. Notably, although many studies have identified neurotrophic effects of triptolide, similar results have not been reported in studies related to celastrol.

The pathogenesis of neurological diseases is complex and not yet fully elucidated. However, studies have shown that neuroinflammation is involved in the development of neurological diseases and that excessive neuroinflammation exacerbates the severity of the diseases (Ransohoff, 2016). Triptolide and celastrol, two important bioactive components extracted from TWHF, play an important role in attenuating neuroinflammation, thus reducing the severity of neurological diseases. In addition to neuroinflammation, triptolide and celastrol also exert neuroprotection against neurological diseases *via* neurotrophic effects and other mechanisms. In this process, regulation of neurons, microglia and astrocytes is involved (Figures 2, 3). Considering the important role of neuroinflammation and neurotrophy and the existence of numerous related studies, we will next review the studies about neuroinflammation and neurotrophy first, and finally other related mechanisms of neuroprotection.

### 5.1 Effects of triptolide and celastrol on neuroinflammation

Inflammation is the body’s response to endogenous and exogenous stimuli and it is important for the body’s self-protection. However, excessive inflammation can lead to dysregulation of inflammation and aggravate the severity of the diseases (Leite et al., 2022). Neuroinflammation has an important role in the pathophysiology of neurological diseases, and triptolide and celastrol have been reported to play a role in attenuating neuroinflammation and thus alleviating neurological diseases. For example, in *vivo* experiments, triptolide and celastrol inhibited neuroinflammation and attenuated the loss of dopaminergic neurons, thus improving the clinical performance of PD animal models (Zhou et al., 2005; Zhang et al., 2021). Further studies revealed that these effects were associated with the inhibition of microglial activation (Li G. et al., 2006). Microglia are innate immune cells of the CNS and are involved in the regulation of neuroinflammation (Young and Denovan-Wright, 2021). Studies have shown that triptolide and celastrol can inhibit the expression of pro-inflammatory molecules (TNF-α, IL-1β, MHC II, COX-2, and prostaglandin E2 (PGE2)) and promote the expression of anti-inflammatory cytokines (IL-4, IL-10 and IL-33) in microglia (Allison et al., 2001; O'Brien et al., 2001; Jiao et al., 2008; Jiang et al., 2018; Sun et al., 2018). Many signaling pathways mentioned have been found to be involved in the process (shown in Figures 2, 3). Among these, the NF-kB signaling pathway, a classical inflammation-related signaling pathway, has been the focus of much research. For example, in animal models of MS, triptolide and celastrol have been reported to reduce neuroinflammation and thereby improve behavioral deficits in model animals by inhibiting NF-kB activity (O'Brien et al., 2001; Wang et al., 2008). In addition to neurodegenerative diseases, the inhibition of NF-kB activity by triptolide and celastrol has also been reported in studies on cerebral ischemia and spinal cord injury (Bai et al., 2016a; Huang et al., 2019; Liu D. D. et al., 2021). In these studies, the inhibition of NF-kB activity will eventually lead to the decrease in the expression levels of pro-inflammatory molecules, while the related studies about downstream of NF-kB are not clear. A study using SCI rat models and LPS + ATP-induced BV2 cells showed that celastrol inhibits the activity of NF-kB, which in turn inhibits the expression of its downstream, NLRP3 inflammasome-related pathway, exerting anti-inflammatory effects (Dai et al., 2019). This reveals that the inhibition of NF-kB activity by celastrol leads to the decrease in the level of pyroptosis, thereby reducing the severity of the disease. In addition, many studies have reported the effects of triptolide and celastrol on Hsp70. Heat shock proteins are molecular chaperones that can assist in the proper folding of newly synthesized proteins and increase induced by stress (Agashe and Hartl, 2000). Kizelsztein et al. found that triptolide increased the expression level of Hsp70, stabilized the NF-kB/IkBα complex, which in turn inhibited neuroinflammation and exerted neuroprotective effects on the EAE models (Kizelsztein et al., 2009). Furthermore, by using LPS-stimulated PC12 cells, Geng et al. found that triptolide inhibited NF-kB activity and phosphorylation of p38, ERK1/2 and AKT, thus reducing the expression of COX-2 and PGE2 (Geng et al., 2012). This shows that in terms of inhibiting neuroinflammation, in addition to targeting microglia, triptolide can also directly target neurons and reduce the level of inflammation in neurons. Similar results can also be found in related studies of celastrol. For example, it was found that celastrol inhibited NF-kB activity and attenuated inflammation levels in HEK293 cells and LPS-treated H4-APP cells (Paris et al., 2010; Zhao et al., 2014). Moreover, in OGD-treated rat primary cortical neurons, celastrol was reported to directly bind to HMGB1 and inhibit NF-kB activity, thus exerting anti-inflammatory effect (Liu D. D. et al., 2021). The above studies demonstrate that triptolide and celastrol can exert neuroprotective effects on neurological diseases by inhibiting neuroinflammation, and their inhibition of neuroinflammation can be achieved by targeting both microglia and neurons.

### 5.2 Effects of triptolide on neurotrophy

In addition to inhibiting neuroinflammation, neurotrophy is also important to achieve the neuroprotective effects of TWHF extracts on neurological diseases. A previous study reported that triptolide promotes the synthesis and release of NGF from rats primary astrocytes (Xue et al., 2007). This suggests that triptolide can exert neurotrophic effects by affecting astrocytes. However, it did not reveal that whether triptolide can affect astrogliosis and glial scars. Astrocytes are important glial cells within the CNS that can play supportive and nutritional roles for neurons. In the case of injury, astrocyte activation leads to astrogliosis and eventually glial scars formation (Nash et al., 2011). In the early stages of injury, the glial scars separate the injured tissue from the surrounding area and protect the spared tissue (Rolls et al., 2009). However, in the late stage, glial scars prevent axonal regeneration and impair the recovery of function (Yiu and He, 2006). Therefore, it is important to study the effects of triptolide on astrogliosis and glial scar in the injured area. Further study using SCI rat models and LPS-stimulated primary astrocytes revealed that triptolide can inhibit astrogliosis and glial scars formation by affecting the JAK2/STAT3 pathway, which in turn promotes spinal cord repair (Su et al., 2010). Moreover, triptolide and tripchlorolide were also found to increase synaptophysin expression in hippocampal neurons, or *BDNF* mRNA expression in primary mesencephalic neurons (Li et al., 2003; Nie et al., 2012). Recently, an *in vivo* study found that triptolide and tripchlorolide increased the expression of neuroligin-1 in the hippocampal region of AD murine models (Lu et al., 2019). These data demonstrate the neurotrophic effects of triptolide in neurological diseases; however, no neurotrophic effect of celastrol has been reported in neurological diseases, and further research is needed.

### 5.3 Effects of triptolide and celastrol on neuroprotection

Besides that, triptolide and celastrol can also exert neuroprotection against neurological diseases through other mechanisms. Polypeptides misfolding is considered as an important pathogenetic mechanism in neurodegenerative diseases (Sweeney et al., 2017). As mentioned earlier, heat shock proteins act as important molecular chaperones to assist in the proper folding of newly synthesized polypeptides. Therefore, targeting heat shock proteins is one of the effective approaches to treat neurodegenerative diseases. It was found that celastrol increases the expression of Hsp70 in neurons, which in turn exerts neuroprotective effects in neurodegenerative diseases (Cleren et al., 2005; Zhang and Sarge, 2007; Kalmar and Greensmith, 2009; Zhao et al., 2014). Besides that, celastrol was also found to increase the expression of Hsp70 in TBI mouse models and SCI spinal cords models, thus exerting neuroprotective effects (Eroglu et al., 2014; Petrović et al., 2019). In addition to increasing the expression of Hsp70, triptolide and celastrol can also exert neuroprotective effects through the following mechanisms: a). decreasing level of oxidative stress. He et al. reported that triptolide inhibited the formation of ROS and the decrease of mitochondrial membrane potential in glutamate-treated PC12 cells (He et al., 2003), b). inhibiting apoptosis. Triptolide inhibits apoptosis by activating the PI3K/AKT/mTOR signaling pathway (Li et al., 2015), c). inducing autophagy. It was reported that triptolide exerts neuroprotection by inducing autophagy in Aβ 25-35-treated differentiated PC12 cells (Xu et al., 2015). Besides that, in MN9D cells, triptolide was found to enhance autophagy in neuronal cells, thus promoting the clearance of α-synuclein (Hu et al., 2017). Moreover, research has shown that celastrol induces autophagy and exerts neuroprotection in rotenone-induced SH-SY5Y cells (Deng et al., 2013), d). attenuating glycolysis. Celastrol attenuates glycolysis and exerts neuroprotection by inhibiting HIF-1α/PDK1 pathway (Chen et al., 2022), e). suppressing NOX activation and rapid H_2_O_2_ release. Celastrol inhibits NOX activation and prevents fast H_2_O_2_ release, which in turn attenuates epileptic seizure (Malkov et al., 2019). Several pathways involved in the neuroprotective effects are shown in Figures 2, 3.

## 6 Toxicity and derivatives

**FIGURE 4:**
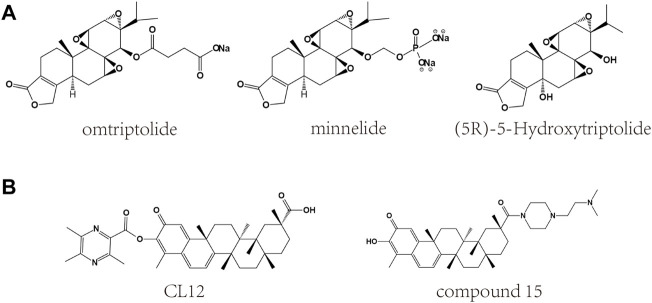
Chemical structures of derivatives of triptolide and celastrol. **(A)** Chemical structures of derivatives of triptolide. **(B)** Chemical structures of derivatives of celastrol.

Although extracts of TWHF can be used to treat a variety of diseases, their toxicity limits clinical application (Li et al., 2014). Among them, triptolide and celastrol have been reported to have hepatotoxicity, nephrotoxicity, cardiotoxicity, reproductive toxicity and hematological toxicity (Table 2) (Bai et al., 2021; Cheng et al., 2021). In addition, poor solubility and bioavailability have also limited the clinical application of triptolide and celastrol, and research on related derivatives (Figure 4) and nanotechnology-based carrier system could improve this situation.

**TABLE 2:** Toxicity of triptolide and celastrol.

Component	Toxicity	*In vivo*/*In vitro*	Effects and mechanisms	References
Triptolide	Hepatotoxicity	*in vivo*; wistar rats	causes necrosis of hepatocytes with inflammatory cell infiltration, involving alteration of hepatic redox status and reduction of serum glucose	Wang et al. (2013a)
		*in vivo*; SD rats	inhibits mitochondrial respiratory chain, thus impairing secondary β-oxidation, which is involved in liver injury	Fu et al. (2011)
		*in vitro*; human liver L02 cells	induces hepatotoxicity, which is associated with mitochondrial fission-associated mitophagy	Hasnat et al. (2019)
	Nephrotoxicity	*in vivo*; SD rats	impairs the antioxidant system and induces oxidative stress, which contributes to nephrotoxicity	Yang et al. (2012)
		*in vivo* and *vitro*; wistar rats, HK-2 and HEK-293T cells	causes nephrotoxicity, involved the expression of organic cation transporter 2 (which transported more TP into kidney cortex)	Shen et al. (2019)
	Reproductive toxicity	*in vivo*; male mice	disrupts testicular structure and inhibit spermatogenesis, which may be associated with downregulation of PPAR and abnormal energy and lipid metabolism	Ma et al. (2015)
		*in vivo*; female mice	decreases the expression levels of estradiol and progesterone, and the cyclic adenosine monophosphate/protein kinase A pathway may be involved	Zhang et al., 2012a; Zhang et al., 2012b
Celastrol	Hepatotoxicity	*in vivo*; SD rats	causes liver damage, and it may be related to the inhibition of the activity of cytochrome P450s	Sun et al. (2014)
		*in vitro*; rat primary hepatocytes	induced hepatotoxicity mediated by hepatic CYP450s	Jin et al. (2019)
	Cardiotoxicity	*in vivo*; zebrafish embryos	results in severe edema in the pericardial sac	Wang et al. (2011)
		*in vivo*; HEK 293 cells	reduces potassium conductance (QT prolongation) by inhibiting K+ channels (hERG and Kir2.1) activity	Sun et al. (2006)
	Hematological toxicity	*in vivo*; BALB/c mice	regulates the hematopoietic cell subsets, thus impairing the development of erythrocytes and B cells	Kusy et al. (2012)

### 6.1 Toxicity

Triptolide is one of the important TWHF extracts and is widely used in therapeutic studies for a variety of diseases. However, it has some toxicity and may cause serious side effects, which limits its clinical application. Its toxicity may be related to the epoxide structure covalently binding to and breaking biological macromolecules in the body (Xu et al., 2013). The liver, as an important metabolic organ in the body, has received widespread attention in the toxic damage caused by triptolide. It was found that the administration of triptolide (1 mg/kg) in animal models caused necrosis of hepatocytes with inflammatory cell infiltration, suggesting a possible role of inflammation in triptolide-induced liver injury (Wang et al., 2013a). This hypothesis is supported by another study which reported liver damage in animal models following triptolide administration, as well as inhibition of mitochondrial respiratory chain and increased levels of oxidative stress (Fu et al., 2011). In addition, it has been suggested that mitochondrial dysfunction and mitochondrial autophagy may also be involved in triptolide-induced liver injury (Hasnat et al., 2019). In addition to hepatotoxicity, nephrotoxicity of triptolide has also been reported. An *in vivo* experiment found that the administration of triptolide (1 mg/kg) to rats induced renal damage and increased level of oxidative stress (Yang et al., 2012). Besides that, triptolide-induced kidney damage was also found in a collagen-induced arthritis rat model (Shen et al., 2019). Moreover, triptolide has been reported to cause reproductive toxicity to both male and female reproductive systems. For example, Ma et al. reported that triptolide can disrupt testicular structure and inhibit spermatogenesis in male mice, which may be associated with downregulation of peroxisome proliferator-activated receptor (PPAR), leading to abnormal energy and lipid metabolism (Ma et al., 2015). As for reproductive toxicity in females, triptolide was found to reduce the expression of estradiol and progesterone in female rats, and the cyclic adenosine monophosphate/protein kinase A pathway may be involved (Zhang et al., 2012a; Zhang et al., 2012b).

Celastrol, one of the main bioactive components extracted from TWHF, has anti-inflammatory, antioxidant, and anti-tumour effects and is therefore used in therapeutic studies for many diseases. However, its toxicity has limited further research in the clinical field. A study reported that celsatrol caused liver damage in rats, which may be related to the inhibition of the activity of cytochrome P450s by celsastrol (Sun et al., 2014). Hepatotoxicity of celastrol was also found in an *in vitro* experiment (rat primary hepatocytes) (Jin et al., 2019). Furthermore, celastrol-induced cardiotoxicity has also been reported. Wang et al. reported that severe edema could be found in the pericardial sac after celastrol treatment (1.5 μM) in zebrafish embryos (Wang et al., 2011). Beside that, the cardiotoxicity of celastrol was found in HEK 293 cells, which may be associated with the inhibition of K+ channels (hERG and Kir2.1) activity (Sun et al., 2006). Moreover, Kusy et al. found that celastrol regulated the hematopoietic cell subsets, thus impairing the development of erythrocytes and B cells, and this further caused hematological toxicity in animal models (Kusy et al., 2012).

### 6.2 Derivatives

Considering the toxicity of triptolide and celastrol, many methods have been used to reduce their toxicity for clinical use. Generally speaking, four methods are usually used including the following: 1). structural modifications, 2). change in processing method, 3). change of the method of administration, and 4). combination of other drugs for co-treatment (Wang et al., 2016). Of these, “structural modification” means investigating derivatives of triptolide and celastrol in order to reduce toxicity, increase efficacy and facilitate clinical application. Structure-activity relationships are important in structural modifications. According to the structure-activity relationship, the derivatives of triptolide obtained after structural modification mainly include omtriptolide (PG490-88) (Kitzen et al., 2009), minnelide (Rivard et al., 2014) and (5R)-5-Hydroxytriptolide (LLDT-8) (Guo et al., 2019). In addition, derivatives of celastrol have also been reported. Celastrol is a triterpenoid quinone methide, and the relevant structural modifications are mainly the C-29 carboxyl group and the A/B ring modification (Bai et al., 2021). Studies have reported that these derivatives can reduce toxicity, increase bioavailability and be more beneficial for clinical applications in a variety of diseases (Zheng et al., 2013; Li and Hao, 2019).

### 6.3 Nanotechnology-based carrier system

With the advancement of nanoscience, nano-sized carriers are being used for drug loading and the nanotechnology-based carrier system can improve the effectiveness of triptolide and celastrol and reduce their toxicity. Studies have demonstrated that triptolide can be encapsulated to form triptolide-loaded nanomaterials, thus decreasing excessive accumulation of triptolide in normal tissues and reducing its toxicity. A study used triptolide-loaded liposomes, which linked anti-carbonic anhydrase IX antibody (an enzyme overexpressed on the surface of lung cancer cells) on the surface, to improve the treatment effects of triptolide on lung cancer cells (Lin et al., 2017). In addition, inflammation-targeted amphiphilic galactosyl dextran-retinal nanoparticles were used to encapsulate triptolide and the all-trans retinal, which is not only conducive to the drug delivery, but also can exert the synergistic effect of the drugs, thereby significantly enhancing the anti-inflammatory effect (Li et al., 2020). Similar to triptolide, celastrol can also be loaded by nanomaterials, which in turn reduces toxicity, increases bioavailability and enhances therapeutic efficacy. Studies have shown that celastrol can be loaded by various types of nanomaterials, such as liposomes, lipid nanoparticles, and exosomes, and the nanomaterials loaded with celastrol are used to treat various diseases (Guo et al., 2021). However, studies on nanotechnology-based carrier system of triptolide and celastrol are mainly about cancer therapy, and there are fewer studies related to neurological diseases (Liaw et al., 2021; Geng et al., 2022). Therefore, more studies are needed to confirm the efficacy of triptolide-loaded or celastrol-loaded nanomaterials for neurological diseases.

## 7 Conclusion

Neurological diseases can affect the brain and spinal cord and cause the corresponding clinical symptoms, which place a greater burden on the patient and society. Therefore, the treatment of neurological diseases is urgent and important. The pathogenesis of neurological diseases is complex and most of them are not due to a single factor; therefore, treatment based on a single-factor etiology is ineffective. Traditional Chinese medicine has a long history of medicinal use in China, with a wide range of targets, and is used for the treatment of many diseases. With the development of modern technology, the research of phytochemical research has gained tremendous progress. Extracts of TWHF (traditional Chinese medicine), triptolide and celastrol, have attracted great interest from researchers because of their structural characteristics and multi-target effects, and have been used in preclinical studies for a variety of neurological diseases involving neurodegenerative diseases, brain and spinal cord injuries, and epilepsy.

Although triptolide and celastrol have excellent potential therapeutic effects, poor water solubility, low oral bioavailability, imprecision of mechanism of action and potential toxicity limit their clinical application. Therefore, many methods have been used to address these problem, such as structural modifications, change in processing method, change of the method of administration, combination of other drugs for co-treatment, and the use of nanotechnology-based carrier system. In addition, to date, there have been no clinical trials to validate the therapeutic effects of triptolide and celastrol in neurological diseases to translate preclinical studies into clinical studies. Considering the potent therapeutic effects of triptolide and celastrol in neurological diseases, further studies on the pathogenesis of neurological diseases, and the mechanism of action, structure-activity relationships and pharmacokinetics of triptolide and celastrol would help to enhance their clinical application. Moreover, the development of nanotechnology can also facilitate the full utilization of these compounds.
